# A retrospective comparison between non-conveyed and conveyed patients in ambulance care

**DOI:** 10.1186/s13049-018-0557-3

**Published:** 2018-10-29

**Authors:** Lilian C. M. Vloet, Arjan de Kreek, Emmelieke M. C. van der Linden, Jori J. A. van Spijk, Vince A. H. Theunissen, Maud van Wanrooij, Pierre M. van Grunsven, Remco H. A. Ebben

**Affiliations:** 1 Faculty of Health and Social Studies, Research Department of Emergency and Critical Care, HAN University of Applied Sciences, PO Box 6960, 6503 Nijmegen, GL The Netherlands; 2 Radboud Institute for Health Sciences IQ Healthcare, Radboud University Medical Center, Nijmegen, The Netherlands; 3 Regional Emergency Medical Service Veiligheidsregio Gelderland-Midden, Arnhem, The Netherlands; 4 Regional Emergency Medical Service Veiligheidsregio Gelderland-Zuid, Nijmegen, The Netherlands

**Keywords:** Emergency medical services [MeSH], Non-conveyance

## Abstract

**Background:**

Not all patients where an ambulance is dispatched are conveyed to an emergency department. Although non-conveyance is a substantial part of ambulance care, there is limited insight in the non-conveyance patient population. Therefore, the study aim was to compare demographics, initial on-scene reasons for care, and vital signs between conveyed and non-conveyed patients attended by an ambulance.

**Methods:**

A retrospective study of ambulance runs from 2 EMS regions in the Netherlands in 2016 was performed. For each ambulance run demographics (age, gender and geographical location), initial reasons for care categorised into the ICD-10 classification system, and vital functions or observational scales (according to the national ambulance care protocol) were collected and analyzed.

**Results:**

54.797 ambulance runs met the inclusion criteria, of which 14.383/54.797 (26.2%) resulted in non-conveyance. There was no significant difference in gender, but the non-conveyance group was significantly younger (48.5 (±26.4) years) compared to the conveyance group (60.7 (±22.2) years) (*p* = .000). The most common initial reasons for care for the conveyance group could be classified into chapter-9 diseases of the circulatory system, chapter-19 injury, poisoning and certain other consequences of external causes, and chapter-10 diseases of the respiratory system. The most common reasons for care in the non-conveyance group could be classified into the chapter-9 diseases of the circulatory system, chapter-19 injury, poisoning and certain other consequences of external causes, and -chapter-5 mental, behavioral and neurodevelopmental disorders. The total percentage abnormal vital functions/observation scales between the conveyance (69.5%) and non-conveyance group (58.6%) was significantly different (*p* = .000). 15 out of 17 vital functions/observation scales are significantly different between the conveyance and non-conveyance group.

**Conclusions:**

This study shows that non-conveyed patients are younger, are more likely to be in (highly) rural areas, and more often have initial reasons for care related to mental, behavioral and neurodevelopmental disorders (ICD-10 chapter 5). Although abnormal vital functions/observation scale were more prevalent in the conveyance group, 58.6% of the non-conveyed patients had at least one abnormal vital function/observation scale.

## Background

Emergency Medical Service (EMS) systems have changed substantially throughout the last century [1]. At the start, EMS-systems contained both regulated and unregulated services, as well as trained professionals as untrained civilians. Due to the improvement of healthcare and the changing population structure, EMS-systems developed from being just a conveyance provider for patients to an advanced emergency care provider. The last years, EMS-systems are developing into mobile integrated health care systems where EMS-professionals perform assessments and interventions without conveyance to an ED [2].

Within these developing EMS-systems, ambulance staff increasingly make critical decisions about patient care in complex environments [3]. One of these critical decisions considers conveyance decision-making. An ambulance professional can choose between conveyance to the emergency department (ED), specialist centers (cardiac, neurological, trauma), referral to another healthcare provider, or non-conveyance [3]. The non-conveyance decision is considered as a complex and difficult task that comes with great responsibility [4]. Non-conveyance is defined as “an ambulance dispatched without subsequent hospital contact, including patients registered dead an ambulance deployment as appropriate, where the patient after examination and/or treatment on-scene does not require conveyance with medical staff and equipment to the hospital” [5, 6]. Non-conveyance can be initiated by the ambulance professional (sometimes after consultation of a general practitioner or medical specialist) and the patient and/or his relatives [7].

A recent systematic review on non-conveyance performed by our research group shows that non-conveyance occurs in all types of EMS-systems and non-conveyance rates for general patient populations vary between 3.7 and 93.7% [8]. The amount of non-conveyed patients increases every year [9]. This increase is attributable to insufficient clarity for members of the public which healthcare service is appropriate for their problem, by the introduction of defensive dispatch triage systems and the situation that the emergency medical dispatcher cannot accurately triage severity of the situation and dispatches an ambulance to be safe [9].

The increase of non-conveyed patients urges the need for the development of guidelines, protocols and policy to manage safely these patients safely, and for appropriate use of healthcare resources. These guidelines and protocols are currently lacking [8]. To develop these instruments, insight in entire prehospital patient population is needed. A recent study reports on diagnoses and outcomes of patients conveyed to the hospital by an ambulance [10]. In addition, the review from our research group on non-conveyance provides a first insight in the non-conveyance population and shows that men and women of all ages, and vulnerable patient groups as people who have fallen and patients with hypoglycemia are represented in the non-conveyance population [8]. Furthermore, non-conveyed patients most often had neurological or trauma related reasons for care. Although this systematic review provides a first insight in characteristics of non-conveyed patients, only three studies reported on vital signs of non-conveyed patients and concluded that about 15% of the non-conveyed patients have vital signs outside normal limits. The review recommends further comparison between conveyed and non-conveyed patients, especially on reasons for care and vital signs.

Therefore, the aim of the present study was to compare demographics, initial on-scene reasons for care, and vital signs between conveyed and non-conveyed patients attended by an ambulance.

## Methods

### Design

The study had a retrospective, descriptive design.

### Setting

Ambulance care in the Netherlands is provided by 25 regional EMSs [11]. Ambulance care is dispatched through the emergency medical dispatch center, and can be requested via the national emergency number, or by other healthcare professionals (such as the general practitioner or medical specialist). Dispatch is either guided by the Advanced Medical Priority Dispatch System, digital variant Professional Quality Assurance (AMPDS), or the Dutch Triage Standard. After triage, ambulance care can be dispatched with urgency level A1 (arrival < 15 min), A2 (arrival < 30 min), and B (ordered ambulance transportation). The dispatch center can dispatch a fully equipped ambulance or a solo vehicle (car or motorcycle). Ambulances are staffed with one driver and one registered ambulance nurse; solo vehicles are staffed with one registered nurse. Registered nurses become qualified as an ambulance nurse after following a specific national training course. Ambulance nurses work autonomously and are allowed to make non-conveyance decisions using their national protocol, without direct consultation of an EMS physician. In addition to regular nurse-based ambulance care a helicopter staffed with a nurse and a physician can be dispatched.

This study took place in two different EMSs in the southeastern part of the Netherlands. EMS region Gelderland-Midden provides ambulance care for 668.000 people, EMS region Gelderland-Zuid provides ambulance care for 541.000 people.

### Data collection

Each ambulance run is stored in an EMS database and has an unique identification number. For this study all ambulance runs from both EMSs from 2016 where extracted. We included a whole year to prevent differences or discrepancies induced by specific months or season of the year [12]. From this sample we excluded ambulance responses with (a) urgency level B, (b) without patient contact, (c) for patient transfer from a hospital to another hospital or discharge to another healthcare facility, and (d) patients who were resuscitated. Therefore, the definitive sample consisted of ambulance runs with A1 or A2 urgency level with either patient conveyance to the hospital or non-conveyance.

For each ambulance run in the definitive sample demographics, initial reasons for care, and vital functions or observational scales were collected. The demographic variables involved age, gender and geographical location. Geographic location was divided in five categories, based on home address per km^2^, from highly urban to highly rural. The variable initial reason for care consists of the 22 different chapters of the International Statistical Classification of Diseases and Related Health Problems 10th revision (ICD-10) [13]. Because the involved EMSs used different classification scales, pairs of two independent researchers (EvdL, JvS, VT, MvW) first converted the reasons for care from the ambulance run databases into the chapters from the ICD-10. An extra code was added for reasons for care that were not classifiable in the chapters of the ICD-10. The vital signs and observation scales involved 19 different variables based on the ABCDE-method, and were based on the national protocol which ambulance nurses in the Netherlands use to make their treatment and conveyance decisions [14]. To determine which values were normal or abnormal, cut-off points based on the Dutch national protocol were used. Table 1 shows the different variables with the corresponding cut-off points and codes. To assess the quality of the data, a random sample of 100 ambulance runs was checked on conversion and accurate cut-off points by two independent researchers. This quality check revealed no systematic errors. Refer to Table 1.

**Table 1:** variables and cut-off points

Variable	Cut-off point	Codes
Demographics		
Age	N/A	Years
Gender		1. Male 2. Female
Geographical location	N/A	1. Highly urban (≥2.500 home address per km^2^) 2. Urban (1.500–2.500 home address per km^2^) 3. Average urban (1.000–1.500 home address per km^2^) 4. Rural (500–1.000 home address per km^2^) 5. Highly rural (≤500 home address per km^2^)
Initial diagnosis		
ICD-10		1. Certain infectious and parasitic diseases 2. Neoplasms 3. Diseases of the blood and blood-forming organs and certain disorders involving the immune mechanism 4. Endocrine, nutritional and metabolic diseases 5. Mental, Behavioral and Neurodevelopmental disorders 6. Diseases of the nervous system 7. Diseases of the eye and adnexa 8. Diseases of the ear and mastoid process 9. Diseases of the circulatory system 10. Diseases of the respiratory system 11. Diseases of the digestive system 12. Diseases of the skin and subcutaneous tissue 13. Diseases of the musculoskeletal system and connective tissue 14. Diseases of the genitourinary system 15. Pregnancy, childbirth and the puerperium 16. Certain conditions originating in the perinatal period 17. Congenital malformations, deformations and chromosomal abnormalities 18. Symptoms, signs and abnormal clinical and laboratory findings, not elsewhere classified 19. Injury, poisoning and certain other consequences of external causes 20. External causes of morbidity 21. Factors influencing health status and contact with health services 22. Codes for special purposes 23. Other/non classifiable
Vital functions & observation scales		
Airway	1. Airway obstructed 2. Airway free	1. Abnormal 2. Normal
Breathing	1. Insufficient breathing 2. Sufficient breathing	1. Abnormal 2. Normal
Respiratory rate (/min.)	Adults (> 12 years) 1. < 12/min. 2. 12–20/min. 3. > 20/min.	1. Abnormal (too low) 2. Normal 3. Abnormal (too high)
	Children < 1 year 1. < 30 min. 2. 30–40/min. 3. > 40/min.	1. Abnormal (too low) 2. Normal 3. Abnormal (too high)
	Children 1–2 years 1. < 25 min. 2. 25–30/min. 3. > 30/min.	1. Abnormal (too low) 2. Normal 3. Abnormal (too high)
	Children 3–5 years 1. < 25 min. 2. 25–30/min. 3. > 30/min.	1. Abnormal (too low) 2. Normal 3. Abnormal (too high)
	Children 6–12 years 1. < 20 min. 2. 20–25/min. 3. > 25/min.	1. Abnormal (too low) 2. Normal 3. Abnormal (too high)
	Children < 1 year 1. < 30 min. 2. 30–40/min. 3. > 40/min.	1. Abnormal (too low) 2. Normal 3. Abnormal (too high)
Oxygen saturation (%)	1. < 96% 2. 96–100%	1. Abnormal (too low) 2. Normal
Carbon dioxide level (kPa)	1. < 4,5 kPa 2. 4,5 kPa – 6,0 kPa 3. > 6,0 kPa	1. Abnormal (too low) 2. Normal 3. Abnormal (too high)
Circulation	1. Insufficient 2. Sufficient	1. Abnormal 2. Normal
Heart rate (/min.)	Adults (> 12 years) 1. < 60/min. 2. 60–100/min. 3. > 100/min.	1. Abnormal (too low) 2. Normal 3. Abnormal (too high)
	Children < 1 year 1. < 110/min. 2. 110–160/min. 3. > 160/min.	1. Abnormal (too low) 2. Normal 3. Abnormal (too high)
	Children 1–2 years 1. < 100/min 2. 100–150/min. 3. > 150/min.	1. Abnormal (too low) 2. Normal 3. Abnormal (too high)
	Children 3–5 years 1. < 95/min. 2. 95–140/min. 3. > 140/min.	1. Abnormal (too low) 2. Normal 3. Abnormal (too high)
	Children 6–12 years 1. < 80/min. 2. 80–120/min. 3. > 120/min.	1. Abnormal (too low) 2. Normal 3. Abnormal (too high)
Systolic blood pressure (mmHg)	Adults (> 12 years) 1. < 90 mmHg 2. 90–160 mmHg 3. > 160 mmHg	1. Abnormal (too low) 2. Normal 3. Abnormal (too high)
	Children < 1 year 1. < 70 mmHg 2. 70–90 mmHg 3. > 90 mmHg	1. Abnormal (too low) 2. Normal 3. Abnormal (too high)
	Children 1–2 years 1. < 80 mmHg 2. 80–95 mmHg 3. > 95 mmHg	1. Abnormal (too low) 2. Normal 3. Abnormal (too high)
	Children 3–5 years 1. < 80 mmHg 2. 80–100 mmHg 3. > 100 mmHg	1. Abnormal (too low) 2. Normal 3. Abnormal (too high)
	Children 6–12 years 1. < 90 mmHg 2. 90–110 mmHg 3. > 110 mmHg	1. Abnormal (too low) 2. Normal 3. Abnormal (too high)
Temperature (°C)	1. < 36,1 °C 2. 36,1–38,0 °C 3. > 38,0 °C	1. Abnormal (to low) 2. Normal 3. Abnormal (too high)
Glasgow Coma Scale (EMV)	1. EMV < 15 2. EMV 15	1. Abnormal 2. Normal
AVPU Scale	1. One of the following: Verbal, Unresponsive, Pain 2. Alert	1. Abnormal 2. Normal
Pupillary response	1. Unequal and non-reactive to light 2. Equal And Reactive to Light	1. Abnormal 2. Normal
Blood glucose level	1. < 3,5 mmol/L. 2. 3,5–14,0 mmol/L. 3. > 14,0 mmol/L.	1. Abnormal (too low) 2. Normal 3. Abnormal (too high)
Disability	1. Episode of unconsciousness 2. Conscious	1. Abnormal 2. Normal
Revised Trauma Score (RTS)	Adults (> 12 years) 1. < 12 2. 12	1. Abnormal (too low) 2. Normal
Pediatric Trauma Score (PTS)	Children (< 12 years) 1. < 12 2. 12	1. Abnormal (too low) 2. Normal
Pain (NRS)	1. NRS 4–10 2. NRS 0–3	1. Abnormal 2. Normal
Sinus rhythm	1. Not present 2. Present	1. Abnormal 2. Normal

### Data analysis

Data were analyzed with SPSS version 24.0 descriptive techniques. To compare the variables between the conveyance group and the non-conveyance group ×^2^-tests, Cramer’s V, and t-tests were performed. Statistical significance was set at *p*-value < 0.05. Results are presented in frequencies and cross-tabulation tables. For the initial reasons for care we reported the top ten ICD-chapters with the highest total incidence, as other groups were too small (< 1.0%).

## Results

**Fig. 1:**
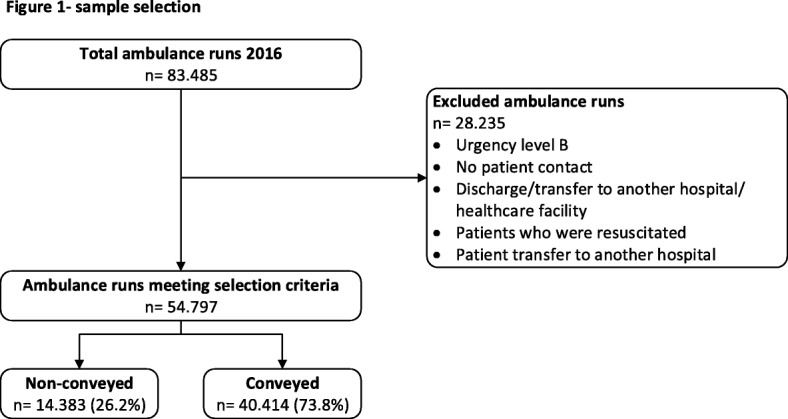
Sample selection

In total, 54.797 ambulance runs met the inclusion criteria, of which 14.383/54.797 (26,2%) resulted in non-conveyance (Fig. 1).

For 53.538/55.797 ambulance runs patient gender was available. As shown in Table 2, there was no significant difference in the proportion of men and women between conveyed an non-conveyed patients. Geographical location was available in 52.419 ambulance runs. The within-group distribution of location was comparable for the conveyance and non-conveyance group, with no patients in highly urban areas, and one third of the patients in urban, average urban and rural areas. The between-group distribution for the non-conveyance and non-conveyance group was significantly different, with the highest difference for the rural location (2.8%). The average age was significantly different between the non-conveyance and conveyance group: 48.5 (±26.4) years for the non-conveyance group, compared to 60.7 (±22.2) years for the conveyance group.

**Table 2:** Demographic characteristics

Variable	Total	Non-conveyance	Conveyance	Difference	*p*-value
Gender n (%)	53,538	13,162	40,376		
Male	26,866	6642 (50.5)	20,224 (50.0)	+ 0.5	.456
Female	26,672	6520 (49.5)	20,152 (50.0)	−0.5	
Geographical location	52,419	12,321	40,098		
Highly urban (≥2.500 home addresses/km^2^)	0	–	–		
Urban (1.500–2.500 home addresses/km^2^)	14,105	3230 (26.2)	10,875 (27.1)	−0.9	.000
Average urban (1.000–1.500 home addresses/km^2^)	14,734	3468 (28.1)	11,266 (28.1)	0.0	
Rural (500–1.000 home addresses/km^2^)	13,289	2856 (23.2)	10,433 (26.0)	−2.8	
Highly rural (< 500 home addresses/km^2^)	4233	826 (6.7)	3407 (8.5)	−1.8	
Age years (±SD)	57.7 (±23.9)	48.5 (±26.4)	60.7 (±22.2)	12.2	.000

The most common reasons for care for the total group and the conveyance group could be classified into chapter-9 diseases of the circulatory system, chapter-19 injury, poisoning and certain other consequences of external causes, and chapter-10 diseases of the respiratory system (see Table 3. The most common reasons for care in the non-conveyance group could be classified into the chapter-9 diseases of the circulatory system, chapter-19 injury, poisoning and certain other consequences of external causes, and chapter-5 mental, behavioral and neurodevelopmental disorders. Between the non-conveyance and conveyance group, all differences were significant, except the difference for chapter-11 diseases of the digestive system.

**Table 3:** Initial diagnosis

Variable	Total (*n* = 54.797) (%)	Non-conveyance (*n* = 14.383)	Conveyance (*n* = 40.414)	Difference	*p*-value
Chapter					
Diseases of the circulatory system (Chapter 9)	13,671 (24.9)	2732 (19.0)	10,939 (27.1)	−8.1	.000
Injury, poisoning and certain other consequences of external causes (Chapter 19)	7410 (13.5)	1753 (12.2)	5657 (14.0)	−1.8	.000
Diseases of the respiratory system (Chapter 10)	3598 (6.6)	198 (1.4)	3400 (8.4)	−7.0	.000
Other/non classifiable	3547 (6.5)	1088 (7.6)	2459 (6.1)	+ 1.5	.000
Symptoms, signs and abnormal clinical and laboratory findings, not elsewhere classified (Chapter 18)	2443 (4.5)	473 (3.3)	1970 (4.9)	−1.6	.000
Diseases of the nervous system (Chapter 6)	1988 (3.6)	439 (3.1)	1549 (3.8)	−0.7	.000
Diseases of the digestive system (Chapter 11)	1756 (3.2)	449 (3.1)	1307 (3.2)	−0.1	.525
Certain infectious and parasitic diseases (Chapter 1)	1661 (3.0)	140 (1.0)	1521 (3.8)	−2.8	.000
Mental, Behavioral and Neurodevelopmental disorders (Chapter 5)	1358 (2.5)	738 (5.1)	620 (1.5)	+3.6	.000
Diseases of the skin and subcutaneous tissue (Chapter 12)	699 (1.3)	275 (1.9)	424 (1.0)	+ 0.9	.000

For 50.402/54.797 (92.0%) of the patients data on vital functions/observation scales was available (see Table 4). Of these, 33.759 (67,0%) had one or more abnormal vital functions. For 38.802/40.414 (96.0%) of the conveyed patients vital functions were available, 26.958/38.802 (69,5%) had one or more abnormal vital functions/observation scales. For 11.600/14.383 (80.7%) of the non-conveyed patients vital functions were available, 6801/11.600 (58,6%) had one or more abnormal vital functions/observation scales. The total percentage abnormal vital functions/observation scales between the conveyance (69,5%) and non-conveyance group (58,6%) is significantly different at *p* = .000 (×^2^ = 475,026, df = 1).

**Table 4:** Abnormal vital functions/observation scales

Variable	Total group Abnormal/registered (%)	Non-conveyance group Abnormal/registered (%)	Conveyance group Abnormal/registered (%)	Difference	*p*-value
Obstructed airway	953/45069 (1.7)	274/10683 (2.6)	679/34386 (2.0)	+ 0.6	.000
Insufficient breathing	3955/44029 (7.2)	523/10482 (5.0)	3432/33547 (10.2)	−5.2	.000
Respiratory rate					.000
Too low	1133/32974 (2.1)	443/7214 (6.1)	690/25760 (2.7)	+3.4	
Too high	7540/32974 (13.8)	1069/7214 (14.8)	6471 (25.1)	−10.3	
Oxygen saturation < 96%	11,246/41699 (20.5)	943/8382 (11.3)	10,303/33317 (30.9)	−19.6	.000
Carbon dioxide level					.000
Too low	447/614 (72.8)	175/208 (84.1)	272 /406 (67.0)	+ 14.1	
Too high	24/614 (3.9)	4/208 (1.9)	20/406 (4.9)	−3.0	
Insufficient circulation	2332/41280 (5.6)	370/9924 (3.7)	1962/31356 (6.3)	−2.6	.000
Heart rate					.000
Too low	2631/46292 (5.7)	517/9814 (5.3)	2114/36478 (5.8)	−0.5	
Too high	9759/46292 (21.1)	1231/9814 (12.5)	8528/36478 (23.4)	− 10.9	
Systolic blood pressure					.000
Too low	1266/41842 (3.0)	172/8092 (2.1)	1094/33750 (3.2)	−1.1	
Too high	9750/41842 (23.3)	1379/8092 (17.0)	8371/33750 (24.8)	−7.8	
Temperature					.000
Too low	1764/11106 (15.9)	411/2364 (17.4)	1353/8742 (15.5)	+ 1.9	
Too high	2592/11106 (23.3)	315/2364 (13.3)	2277/8742 (26.0)	−12.7	
Glasgow Coma Scale (EMV) < 15	5106/44657 (11.4)	870/9794 (8.9)	4236/34863 (12.2)	−3.3	.000
Abnormal AVPU scale	3927/39889 (9.8)	826/9658 (8.6)	3101/30231 (10.3)	−1.7	.000
Abnormal pupillary response	363/23502 (1.5)	80/5919 (1.4)	283/17583 (1.6)	−0.2	.181
Blood glucose level					.000
Too low	494/18646 (2.6)	251/3683 (6.8)	243/14963 (1.6)	+ 5.2	
Too high	3735/18646 (20.0)	533/3683 (14.5)	3202/14963 (21.4)	−6.9	
Abnormal disability	1411/4648 (30.4)	327/1270 (25.7)	1084/3378 (32.1)	−6.4	.000
RTS/PTS < 12	4180/25367 (16.5)	491/5237 (9.4)	3689/20130 (18.3)	−8.9	.000
Pain score (NRS) > 3	3706/6640 (55.8)	251/876 (28.7)	3455/5764 (59.9)	−31.2	.000
Non-sinus rhythm	20,553/39458 (52.1)	4743/9240 (51.3)	15,810/30218 (52.3)	−1.0	.098

Table 4 shows that 15/17 vital functions/observation scale are significantly different between the conveyance and non-conveyance group. Of these 15, an obstructed airway (2.6% vs. 2.0%), too low respiratory rate (6.1% vs. 2.7%), too low CO_2_-rate (84.1% vs. 67.0%), too low temperature (17.4% vs. 15.5%), and too low glucose level (6.8% vs. 1.6%), all were significantly more prevalent in the non-conveyance group than in the conveyance group. Of the vital functions/observation scales that were significantly more prevalent in the conveyance group than in the non-conveyance group, the pain score (28.7% vs. 59.9%), oxygen saturation < 96% (11.3% vs. 30.9%), and too high temperature (13.3% vs. 26.0%) showed the biggest differences.

## Discussion

This study compared demographics, initial on-scene reasons for care, and vital signs between conveyed and non-conveyed patients attended by an ambulance. The results showed that non-conveyed patients are younger, are more likely to be in (highly) rural areas, more often have reasons for care related to mental, behavioral and neurodevelopmental disorders (ICS-10 chapter 5), and more than half of these patients have at least one abnormal vital function/observation scale.

As for demographics, there was no significant difference in gender between conveyed and non-conveyed patients. This is congruent with a recent systematic review on non-conveyance [8]. Possibly, our result can be explained by the demographic composition of the Netherlands where gender is equally distributed. As for age, the non-conveyance group has a significant lower age compared to the conveyance group, this is comparable with previous research [15, 16]. Possibly, this can be explained by the holistic assessment of the patient, where not only the medical condition is taking into account, but also the coping ability [15]. Also, older patients might call an ambulance for an acute exacerbation of a chronic disease [10]. Compared to conveyed patients, there are less non-conveyed patients in the rural and highly rural areas. This result is comparable with findings of the previous systematic review [8]. This might be due to the lack of alternative healthcare facilities in these areas, that limits referral options for ambulance staff. Another explanation might be that people in (highly) rural areas have different socioeconomic characteristics and therefore are less likely to call for medical help [17].

Our study shows a variety of initial reasons for care in the prehospital patient population. Around 40% of the reasons for care for the total group and conveyance group could be classified into chapter-9 diseases of the circulatory system and chapter-19 injury, poisoning and certain other consequences of external causes. This is comparable with a recent study [10]. The non-conveyance population shows a wide variation of on-scene reasons for care comparable with a recent study [18]. Comparing on-scene reasons for care from ambulance nurses, there a significantly more patients with mental, behavioral and neurodevelopmental disorders (chapter-5) in the non-conveyance group compared to the conveyance group. Literature shows contrasting results, with studies reporting 9.0–19.1% psychiatry or alcohol/drugs abuse reasons for care in the non-conveyance group [15, 16, 18], and another study reporting that patients with psychiatric reasons for care were more likely to be conveyed [19]. These patients represent a vulnerable patient group in which it can be questioned if ambulance care is the most appropriate [20], and if ambulance staff currently have the competencies to manage patients with psychological and social problems. Our results indicate that the development of guidelines and alternative care options for these patients is needed, for instance mental health acute assessment teams [21].

Our results show that 58.6% of the patients in the non-conveyance group had at least one abnormal vital function/observation scale. This percentage is high compared to previous research [8]. Possible explanations are that abnormal values can be related (for instance, respiratory rate and O_2_-saturation), might be present in medical history (too high systolic blood pressure), or can be treated on-scene. For instance, hypoglycemia often can be safely treated on-scene without medical follow-up [22], whereas hyperglycemia comes with other risks or is a symptom of other medical conditions. Another reason might be poor registration on vital functions and observation scales for non-conveyance ambulance runs. The poor registration on non-conveyance ambulance runs is recognized in literature [6]. This might be caused by the fact that in case of non-conveyance situations with normal vital signs and observation scales these data are not registered on the ambulance run records. However, from the perspectives of medicolegal and continuity of care, these data should be registered.

Furthermore, there is a significant difference on Airway, Breathing, Circulation, and Disability between the non-conveyance and conveyance group. Abnormal values for Breathing and Circulation seem an indication for conveyance, with a contrast for a too low respiratory rate which is more prevalent in the non-conveyance group. Although most vital functions/observation scales are significantly different between the conveyance and non-conveyance group, abnormal vital functions/observation scales are also present in the non-conveyance group. This indicates that vital functions/observation scale cannot be used alone to make a non-conveyance decision, and that additional decision rules should be incorporated in the current non-conveyance protocols. The non-conveyance decision is multifactorial and complex [8, 23] and has influences from the patient and his relatives, the professional, the healthcare system, and supportive tools. For ambulance staff, this might require additional competences and training [4, 24].

### Limitations

The first limitation is that, due to random suboptimal registration of demographic characteristics, initial reasons for care and vital functions we had missing data on all variables. Missing data in non-conveyance studies has been described earlier [18]. Secondly, our study did not compare patient outcomes and follow-up care between conveyed and non-conveyed patients as this information is not registered on the ambulance run sheet. Although these outcomes are not present for our study population, both EMS regions use the non-conveyance quality indicator ‘the percentage of renewed ambulance contact within 24 hours after non-conveyance’. For 2016 these percentages were 0,29% (EMS region Gelderland-Zuid) and 0,45% (EMS region Gelderland-Midden), which indicates safe non-conveyance care in both regions. A possible third limitation concerns the use of the ICD-10 classification system. Although widely accepted, it is not primary developed for prehospital care. Finally, this study was conducted in the Netherlands with a specific EMS-system where ambulance referral to the ED, general practitioner or medical specialist are options. This might limit the possibility to generalize our results to other healthcare systems.

## Conclusion

This study shows that non-conveyed patients have significantly different demographical characteristics, initial reasons for care and vital functions/observation scales compared to conveyed patients. Non-conveyed patients are younger, and there are less non-conveyed patients in the rural and highly rural areas. There is a variety of initial reasons for care in the entire prehospital patient population. Common reasons for care in the conveyance and non-conveyance group are related to diseases of the circulatory system (chapter-9) and injury, poising and other consequences of external causes (chapter-19). In contrast, there a significantly more patients with mental, behavioral and neurodevelopmental disorders in the non-conveyance group. The conveyed group more often has one or more abnormal vital functions than the non-conveyance group.
